# Tacrolimus-Induced Type IV Renal Tubular Acidosis following Liver Transplantation

**DOI:** 10.1155/2017/9312481

**Published:** 2017-07-06

**Authors:** Christopher Schmoyer, Suraj Mishra, Frank Fulco

**Affiliations:** ^1^Department of Internal Medicine, Virginia Commonwealth University, Richmond, VA, USA; ^2^Department of Internal Medicine, Hunter Holmes McGuire VA Medical Center, Richmond, VA, USA

## Abstract

Calcineurin inhibitors remain an integral component of immunosuppressive therapy regimens following solid organ transplantation. Although nephrotoxicity associated with these agents is well documented, type IV renal tubular acidosis is a rare and potentially underreported complication following liver transplantation. Hepatologists must be able to recognize this adverse effect as it can lead to fatal hyperkalemia. We describe a case of tacrolimus-induced hyperkalemic type IV renal tubular acidosis in a patient following an orthotopic liver transplant for alcoholic cirrhosis.

## 1. Introduction

Calcineurin inhibitors (CNIs) such as cyclosporine and tacrolimus continue to form the backbone of immunosuppressive regimens used in solid organ transplantation [1]. Both acute and chronic nephrotoxicity have been associated with these agents [2]. A case of type IV renal tubular acidosis (RTA), a rare case of acute nephrotoxicity associated with CNI use, is detailed in this report.

## 2. Case Report

A 55-year-old male with a history of decompensated alcoholic cirrhosis and human immunodeficiency virus (HIV) on a fixed dose dolutegravir based regimen (viral load undetectable) underwent an orthotopic liver transplant. Surgery was complicated by intraoperative hypotension resulting in acute tubular necrosis and stage 3 acute kidney injury requiring continuous renal replacement therapy following transplantation. Kidney function returned to baseline by posttransplant day 5 and renal replacement therapy was discontinued. Synthetic function of the liver graft was excellent as noted in laboratory markers of liver failure following transplantation. Aspartate aminotransferase improved from 91 to 20 (15–37 IU/L), alanine aminotransferase from 41 to 72 (12–78 IU/L), alkaline phosphatase from 261 to 157 (45–117 IU/L), total bilirubin from 11.8 to 0.4 (0.2–1.0 mg/dL), albumin from 1.7 to 3.4 (3.4–5.0 g/dL), and platelets from 31 to 120 (130–400 × 10^3^/*μ*L) before and after transplant, respectively. There was no clinical, laboratory, or radiologic evidence of graft rejection, biliary stricture, or other complications.

Immediately following transplantation, an immunosuppressive regimen of mycophenolate mofetil and prednisone was initiated. Tacrolimus was added on posttransplant day 5 following resolution of the perioperative acute kidney injury. Serum tacrolimus levels rose thereafter, peaking on posttransplant day 13 at 14.1 ng/mL. During this period, serum potassium levels steadily elevated from a previous normal range, reaching 6.0 mmol/L (3.5–5.1 mmol/L) on postoperative day 16. An EKG did not demonstrate prolonged PR or QRS intervals nor peaked T waves. Labs were also notable for elevated chloride of 113 mmol/L (98–107 mmol/L), bicarbonate of 19 mmol/L (21–32 mmol/L), and a corrected anion gap of 11.75 (8–15 mmol/L). As the patient was not experiencing any diarrhea, a workup for renal causes of his non-anion gap metabolic acidosis was undertaken. Analysis revealed a positive urine anion gap of 53.7 mEq/L. An aldosterone level obtained at 8 AM the following morning was suppressed at 2 ng/dL (3–16 ng/dL) while renin was inappropriately in the low-normal range at 0.98 ng/L/hr (0.25–5.82 ng/L/hr). These findings were consistent with type IV renal tubular acidosis. The tacrolimus dose was adjusted to achieve a goal concentration of 10–12 ng/mL. However, serum potassium levels remained elevated. As tacrolimus was an integral part of the immunosuppressive regimen, 0.1 mg fludrocortisone daily was added to correct the patient's hypoaldosterone state. The following day, renal parameters improved to normal range with serum potassium 4.5 mmol/L, chloride 107 mmol/L, and bicarbonate 24 mmol/L. Arterial blood gas was also obtained with pH 7.38, pCO_2_ 48, and pO_2_ 78. Potassium remained in the normal range throughout the remainder of this hospitalization.

## 3. Discussion

Calcineurin inhibitors exert their immunosuppressive effects by inhibiting nuclear factor of activated T-cells (NFAT) reducing interleukin-2 transcription and T-cell activation [2]. Blocking NFAT mediated gene transcription in other cell types causes many of the adverse effects associated with this drug class. The most common form of CNI nephrotoxicity develops shortly after drug initiation. Activation of the renin-angiotensin system with reductions in nitric oxide, prostaglandin E2, and prostacyclin leads to vasoconstriction of the afferent arteriole. The effect is a dose dependent and reversible elevation in blood pressure and reduction in glomerular filtration rate [3]. Chronic NFAT downregulation leads to hyalinosis of the renal vasculature, tubular atrophy and fibrosis, and glomerular thickening with either segmental or diffuse sclerosis. These changes occurred in the majority of patients on long-term CNI in a cohort following heart transplantation [4].

Type IV RTA is a rare form of acute nephrotoxicity associated with CNI use. This phenomenon has only been described in rare cases following liver transplantation [5–7]. Patients with type IV RTA tend to be asymptomatic with normal creatinine and urine output. However, the associated hyperkalemia can lead to paralysis or fatal arrhythmia making early diagnosis and proper treatment a priority. A high index of suspicion must be maintained for type IV RTA in the presence of normal anion gap metabolic acidosis with associated hyperkalemia and hyperchloremia. The diagnosis is confirmed by abnormal urine studies demonstrating a positive urine anion gap and, often, acidic urine with pH of <5.5. Levels of renin and aldosterone are also frequently low [8]. In general, the cause of type IV RTA is either low levels of aldosterone or the impaired ability of the renal tubule to respond to this hormone. Known etiologies include primary or secondary adrenal insufficiency, acquired or congenital defects in the epithelial sodium channel, and the adverse effects of medications [9]. Common medications linked to type IV RTA include angiotensin inhibitors, potassium sparing diuretics, and trimethoprim [8]. CNIs lead to type IV RTA by reducing the activity of the Na^+^/K^+^ ATPase as well as the Na^+^/K^+^/2CL^−^ cotransporter [10]. These changes prevent the kidney from maintaining the normal electrochemical gradient, reducing urinary potassium and hydrogen ion secretion. CNI also downregulates gene expression of mineralocorticoid receptors which, through a still unknown mechanism, leads to reductions in serum aldosterone itself [11]. The hyperkalemia associated with CNI induced type IV RTA may improve with dose reductions of the immunosuppressive agent. However, treatment of the underlying hypoaldosterone state with a mineralocorticoid, such as fludrocortisone, is often required [12, 13]. In conclusion, type IV RTA is a rare and underreported complication of tacrolimus use following liver transplantation that must be recognized by hepatologists as it can lead to severe and potentially fatal hyperkalemia.
